# Crystallinity-Regulated Hydration, Molecular Mobility, and Mechanical Softening of Poly(ether ether ketone): A Reactive Molecular Dynamics Study

**DOI:** 10.1021/acsomega.6c08565

**Published:** 2026-10-28

**Authors:** Xiawa Wu

**Affiliations:** Mechanical Engineering, 43312 Penn State Behrend, Erie, Pennsylvania 16563, United States

## Abstract

Poly­(ether ether ketone) (PEEK) is a high-performance semicrystalline thermoplastic widely used in aerospace, biomedical, and energy applications because of its excellent mechanical properties and resistance to harsh environments. However, the molecular mechanisms governing its short-time response to moisture exposure remain incompletely understood. In this work, reactive molecular dynamics simulations were performed to investigate the short-time hydration response of amorphous and semicrystalline PEEK in the dry state and at 1 and 3 wt % water. Water distribution, local hydration structure, molecular mobility, density-derived glass-transition behavior, and constrained directional moduli were analyzed to establish relationships among crystallinity, hydration, and thermomechanical behavior. Water showed greater local association with carbonyl oxygen than with ether oxygen, while increasing water content increased local coordination and molecular fluctuations. In semicrystalline PEEK, crystalline domains spatially confined these hydration-induced mobility enhancements, whereas the density-derived apparent glass-transition temperature showed a modest downward trend at 3 wt % water that was not resolved within the observed simulation variability. Increasing water content nevertheless progressively reduced the constrained directional moduli through plasticization, particularly within the amorphous and crystal–amorphous interfacial regions, whereas the crystalline phase retained substantial chain-direction stiffness. These results indicate that within the constructed models and sampled time scale, crystalline morphology restricted hydration-induced plasticization by limiting water accessibility and molecular mobility. No persistent hydration-induced bond-cleavage events were observed over the simulated time scale.

## Graphical abstract

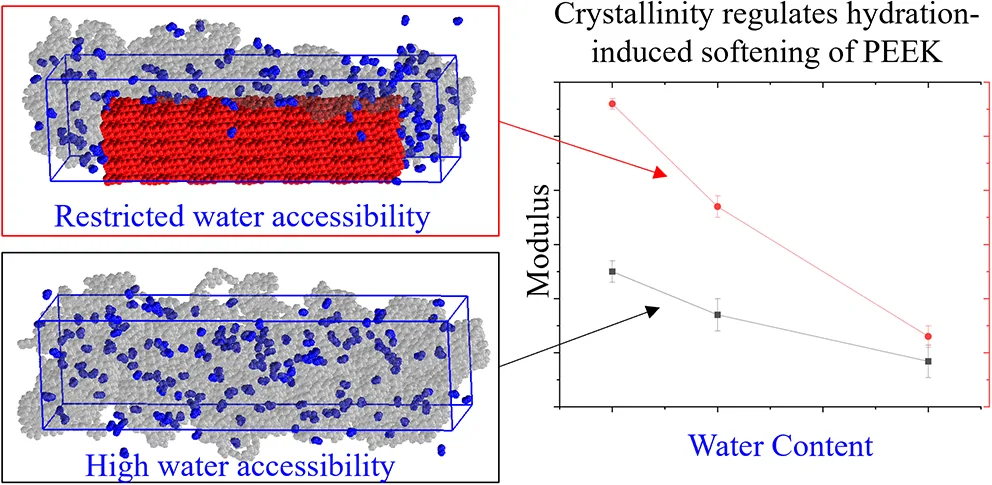

## Introduction

Poly­(ether ether ketone) (PEEK) is a high-performance semicrystalline thermoplastic belonging to the poly­(aryl ether ketone) (PAEK) family. Its molecular backbone, composed of alternating aromatic rings, ether (−O−), and ketone (−CO−) groups, provides a combination of thermal stability, chemical resistance, mechanical strength, and fatigue resistance.^1–3^ With a glass transition temperature of approximately 416 K and a melting temperature near 616 K, PEEK has become one of the most widely used engineering thermoplastics for demanding applications in the aerospace, automotive, oil and gas, and biomedical sectors.^2,3^ In particular, its biocompatibility, radiolucency, and mechanical properties have enabled its extensive use in orthopedic implants, spinal cages, and dental devices, where the material may experience prolonged exposure to physiological fluids and repeated sterilization.^1,4^

Although PEEK is generally regarded as one of the most hydrothermally stable engineering polymers, prolonged exposure to moisture and elevated temperature can gradually alter its mechanical performance. Experimental studies have reported water absorption, plasticization of the polymer matrix, reductions in stiffness, and degradation of fiber–matrix interfaces under hygrothermal conditions.^5–7^ Moisture uptake generally increases with temperature and pressure, accelerating mechanical degradation and reducing the interfacial strength of carbon-fiber-reinforced PEEK composites.^5–7^ Under more aggressive hydrothermal conditions, chain scission and decomposition have also been reported, particularly in the presence of catalysts or subcritical water.^8,9^ Collectively, these observations show that the hydrothermal response of PEEK is condition dependent, ranging from water uptake and physical plasticization to irreversible chemical degradation under sufficiently severe conditions.

Despite extensive experimental investigation, the molecular mechanisms governing the short-time hydration response of PEEK remain poorly understood. In particular, it is unclear how water is organized within amorphous, crystalline, and interfacial environments, how hydration alters local segmental mobility, and how crystalline morphology influences the thermomechanical response. Experimental studies have characterized moisture uptake, mechanical changes, and spectroscopic signatures of hydration in PEEK.^5–7^ Atomistic simulations complement these measurements by resolving water coordination around individual functional groups within amorphous, crystalline, and interfacial environments.

Previous MD studies of PEEK have primarily examined its dry thermomechanical properties or the interfacial behavior of fiber-reinforced PEEK composites.^10–13^ Reactive MD simulations of amorphous and crystalline PEEK have demonstrated that crystallinity strongly affects stiffness and produces pronounced mechanical anisotropy in the crystalline phase.^10,11^ However, the coupled effects of hydration and crystallinity on water distribution, segmental mobility, glass transition behavior, and mechanical properties remain largely unexplored. Consequently, the role of crystalline morphology in the short-time hydration-induced structural and mechanical response of semicrystalline PEEK has not been clearly established.

In this work, reactive molecular dynamics simulations based on the ReaxFF force field^14^ were performed to investigate the short-time hydration response of amorphous and semicrystalline PEEK containing 0 (dry), 1, and 3 wt % water. The reactive force field was selected to resolve local water–PEEK interactions while allowing potential hydration-induced changes in atomic connectivity to be monitored over the simulated interval. Water contents of 1 and 3 wt % were selected as elevated model loadings to compare concentration-dependent hydration and plasticization. The 3 wt % loading represents the upper concentration investigated, not a predicted saturation limit. For context, an experimental study reported approximately 0.32 wt % equilibrium water uptake for neat PEEK at 343 K.^15^ The imposed model loadings therefore do not represent equilibrium uptake at a specific service condition. Regional water populations, radial distribution functions, coordination numbers, mean square fluctuations (MSF), density-derived glass-transition behavior, and constrained directional moduli were analyzed to establish relationships among crystallinity, hydration, molecular mobility, and mechanical response. Direct comparison of the two morphologies was used to determine how crystalline packing restricts water accessibility and limits hydration-induced molecular fluctuations. The results provide molecular-level insight into the short-time physical hydration and plasticization response of semicrystalline PEEK and establish a computational framework for relating local water organization to thermomechanical behavior.

## Results and Discussion

### Effects of Temperature and Hydration on Equilibrium Density

The equilibrium densities of A-PEEK and S-PEEK containing dry, 1, and 3 wt % water are shown in Figure 1. At 300 K, all simulated systems exhibited densities of approximately 1.22–1.25 g cm^–3^ (Figure 1(a)). The simulated A-PEEK density of approximately 1.23 g cm^–3^ was slightly lower than the value of 1.30 ± 0.01 g cm^–3^ reported for ReaxFF-equilibrated amorphous PEEK^10,16^ and the experimental density of 1.262 ± 0.001 g cm^–3^ reported for quenched amorphous PEEK.^17^ The semicrystalline models also underestimated the density expected from their crystalline mass fraction. The density differences were small relative to the variability across the four simulations.

**Figure 1:**
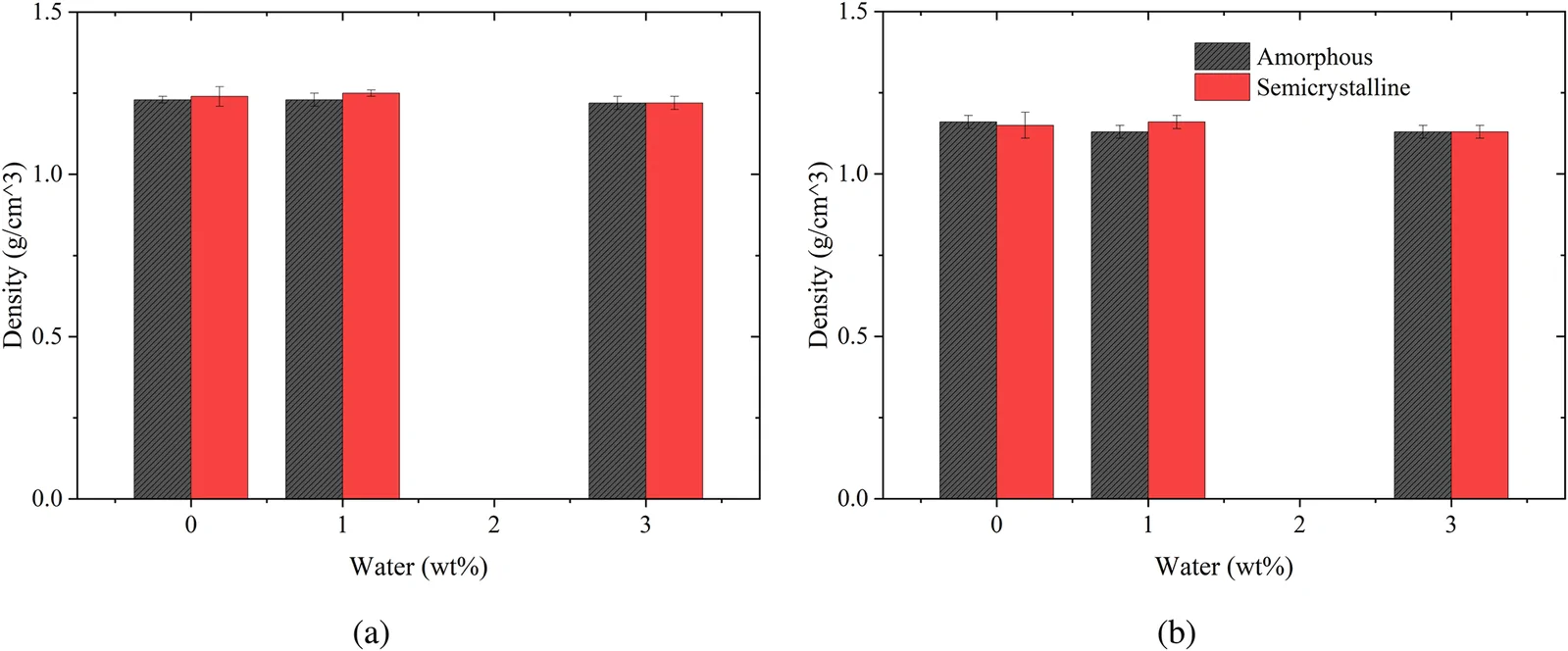
Equilibrium densities of fully amorphous and 36 wt % semicrystalline PEEK containing dry, 1, and 3 wt % water at (a) 300 and (b) 500 K.

At 500 K, the simulated densities decreased to approximately 1.13–1.16 g cm^–3^. Experimental pressure–volume–temperature data indicate a PEEK density of approximately 1.16–1.20 g cm^–3^ near 500 K at low pressure, depending on crystallinity and thermal history.^18^ The upper end of the simulated range is therefore comparable to the experimental estimate, whereas some models exhibit a greater degree of thermal expansion. This reduction reflects thermal expansion and the increased segmental mobility of PEEK at elevated temperature. In contrast, the density differences associated with water content remained small and showed no consistent monotonic trend. Thus, over the conditions examined, temperature was the dominant factor governing the bulk density, whereas the influence of water contents up to 3 wt % was not resolved by this measurement.

### Effect of Hydration on the Glass Transition Temperature

The glass-transition behavior of S-PEEK was evaluated from the temperature dependence of the equilibrium system density, as shown in Figure 2. Separate linear regressions were fitted to the low-temperature glassy regime (200–300 K) and the high-temperature rubbery regime (500–600 K), and the intersection of the two regressions was used to estimate the glass transition temperature, *T*_g_. The intermediate 320–480 K data were excluded because they span the glass-transition region and therefore do not follow the approximately linear thermal-expansion behavior of either the glassy or rubbery regime. The low-/high-temperature regressions yielded *R* ^2^ values of 0.97/0.98, 0.96/0.97, and 0.90/0.96 for the dry, 1, and 3 wt % water systems, respectively. Because the transition arises primarily from changes in segmental mobility within the noncrystalline regions, the resulting value represents the effective glass transition of the amorphous and crystal–amorphous interfacial phases within the semicrystalline model.

**Figure 2:**
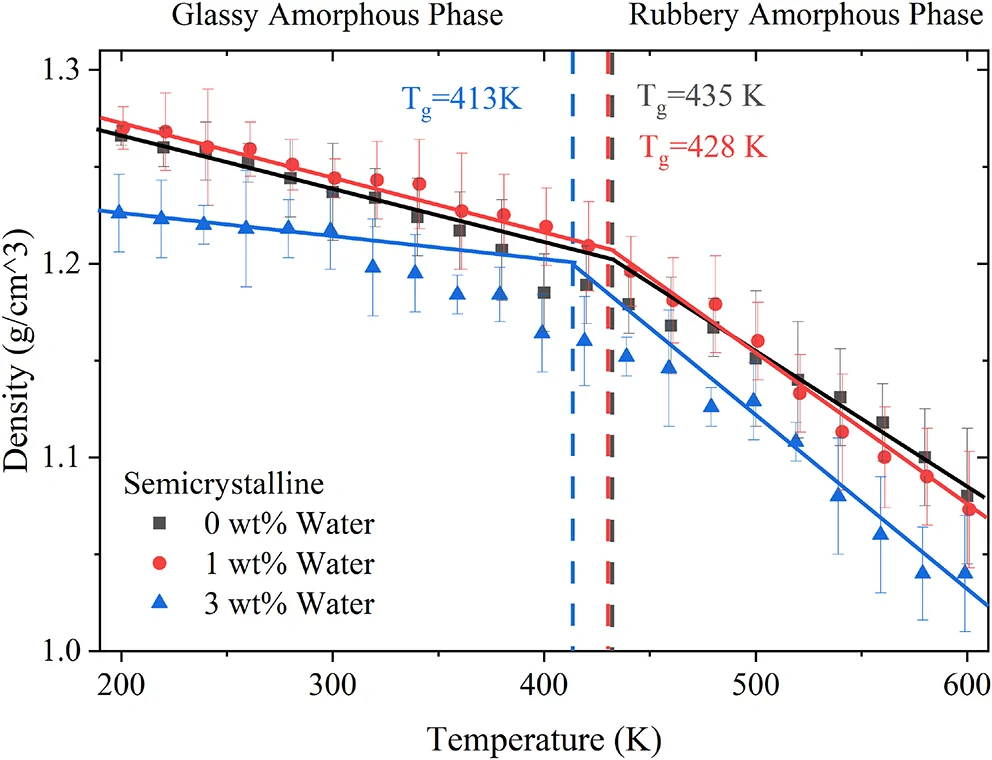
Temperature-dependent equilibrium density of semicrystalline PEEK containing 0 (dry), 1, and 3 wt % water. All models contained approximately 36 wt % crystalline PEEK. Symbols represent the mean densities obtained from four simulations initialized with different velocities, and error bars represent the corresponding standard deviations. Solid lines show linear fits to the low-temperature and high-temperature regimes. The intersection of the fitted lines yielded estimated glass transition temperatures of 435 K, 428 K and 413 K for the dry, 1, and 3 wt % hydrated system, respectively.

The primary regressions yielded apparent *T*_g_ values of approximately 435, 428, and 413 K for dry, 1, and 3 wt % water, respectively. However, uncertainty propagation and fitting-range sensitivity analysis did not resolve the hydration-induced differences. The fitting results and approximate 95% uncertainty intervals are provided in Section S1 of the Supporting Information.

The estimated values are comparable to the experimental glass transition temperature of PEEK, which is commonly reported near 416–418 K (143–145 °C).^19^ Previous atomistic simulations have also predicted PEEK glass-transition temperatures near 424–430 K, although density-derived estimates depend on the force field, chain length, thermal protocol, simulation time scale, replica variability, and selected fitting ranges.^20–22^ The agreement therefore supports the ability of the present model to reproduce the approximate temperature range of the PEEK glass transition.

Although the fitted *T*_g_ decreased with increasing water content, the uncertainty analysis did not resolve a hydration-induced shift. The lower estimate at 3 wt % water is qualitatively consistent with plasticization and the hydration and mobility results discussed below, but those observations do not independently establish a transition-temperature reduction.

### Molecular-Scale Hydration Behavior of PEEK

#### Hydration of Amorphous PEEK

The local distributions of water oxygen atoms around the ether and carbonyl oxygen sites of A-PEEK were characterized using water-oxygen–PEEK-oxygen radial distribution functions and cumulative coordination numbers, as shown in Figure 3. Although both PEEK oxygen sites can act as hydrogen-bond acceptors, these O···O metrics quantify spatial association and do not independently identify hydrogen bonds. The two sites nevertheless exhibited distinctly different local water distributions.

**Figure 3:**
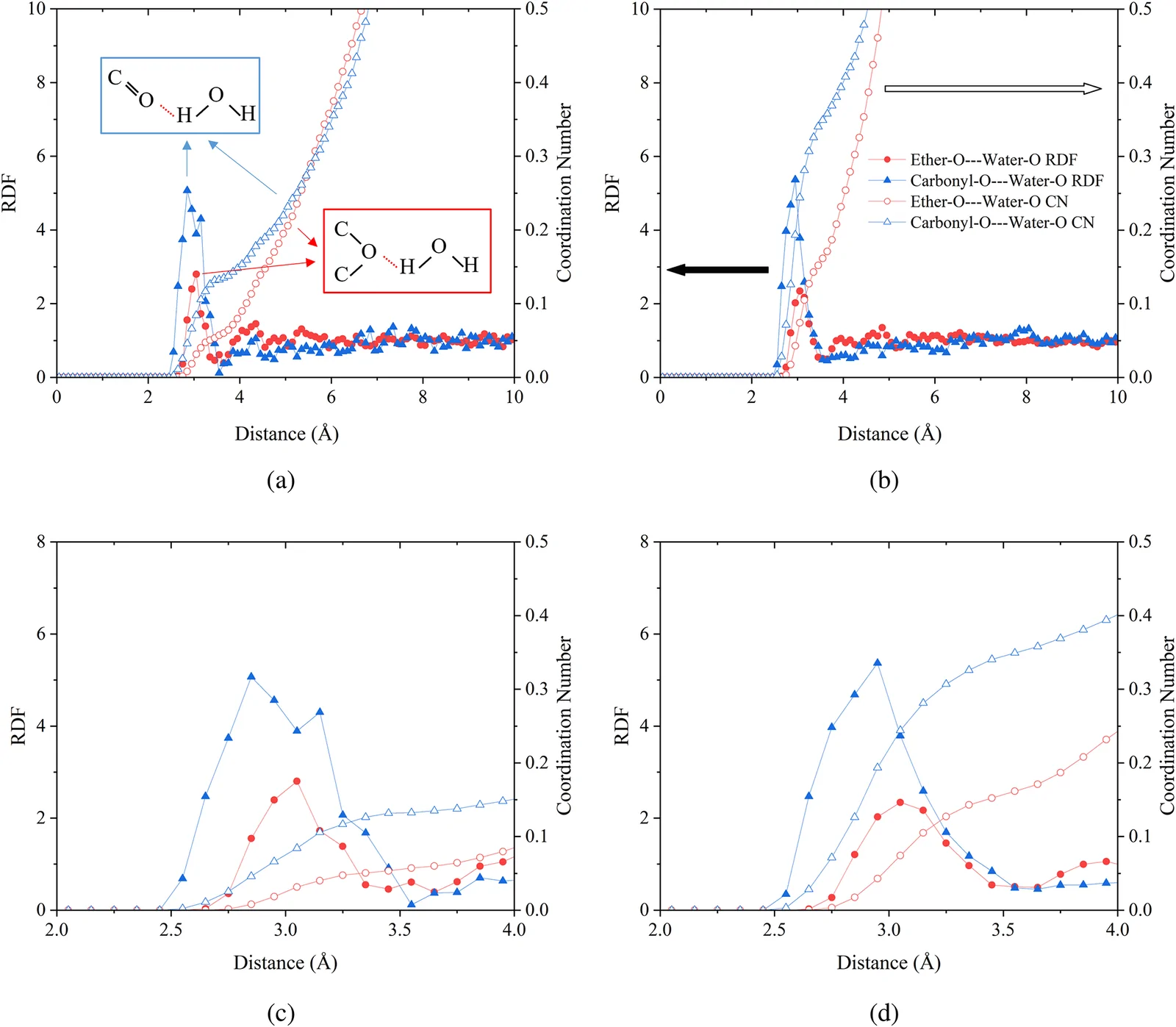
Radial distribution functions (RDFs) and cumulative coordination numbers (CNs) of water oxygen atoms around the ether and carbonyl oxygen atoms of amorphous PEEK containing (a) 1 wt % and (b) 3 wt % water at 300 K. (c, d) show the corresponding 2–4 Å regions of (a, b), respectively. Filled symbols represent RDFs (left axis), and open symbols represent cumulative coordination numbers (right axis). The cumulative CN is the average number of water oxygen atoms within distance *r* of a PEEK oxygen site; first-shell CNs were evaluated at the first minimum of each corresponding RDF.

At both hydration levels, the carbonyl-oxygen–water-oxygen RDF exhibited a pronounced first maximum at 2.85–2.95 Å, whereas the ether-oxygen–water-oxygen RDF showed a lower and broader maximum at approximately 3.05 Å. At 1 wt % water, the first maxima occurred at 2.85 and 3.05 Å for the carbonyl and ether oxygen atoms, respectively. At 3 wt % water, the corresponding peak positions were 2.95 and 3.05 Å. These separations fall within the range commonly associated with hydrogen-bond-compatible O···O contacts.^23^

The substantially larger carbonyl RDF maximum indicates a greater probability of finding water near a carbonyl oxygen than near an ether oxygen at the corresponding first-shell distance. Increasing the water content from 1 to 3 wt % did not appreciably shift either first-shell maximum. Thus, hydration level primarily affected the population of water near the two functional groups rather than their preferred local contact distances.

The first-shell coordination numbers quantify this difference (Table 1). At 1 wt % water, each carbonyl oxygen was surrounded by an average of 0.13 water oxygen atoms within its first coordination shell, compared with 0.05 around each ether oxygen. Carbonyl oxygen therefore exhibited approximately 2.6 times the first-shell water occupancy of ether oxygen. At 3 wt % water, the corresponding coordination numbers increased to 0.34 and 0.15, respectively, retaining a carbonyl-to-ether occupancy ratio of approximately 2.3. These results demonstrate preferential local association of water with the carbonyl groups of amorphous PEEK.

**Table 1:** First-Shell RDF Peak Positions and Coordination Numbers of Water Oxygen Atoms around the Ether and Carbonyl Oxygen Atoms of Amorphous and Semicrystalline PEEK at 300 K^a^

Model, Water, Region	Ether Peak Position (Å)	Ether First-Shell CN	Carbonyl Peak Position (Å)	Carbonyl First-Shell CN
A-PEEK, 1 wt %, Amorphous	3.05	0.05	2.85	0.13
A-PEEK, 3 wt %, Amorphous	3.05	0.15	2.95	0.34
S-PEEK, 1 wt %, Crystalline	2.95	0.02	2.95	0.04
S-PEEK, 1 wt %, Amorphous	3.15	0.08	2.65	0.15
S-PEEK, 3 wt %, Crystalline	3.05	0.06	3.05	0.11
S-PEEK, 3 wt %, Amorphous	3.05	0.19	3.05	0.44

a Peak positions correspond to the first RDF maxima, and first-shell coordination numbers were evaluated at the first minima.

Tripling the water content increased the first-shell coordination number by factors of approximately 2.56 for carbonyl oxygen and 2.98 for ether oxygen. The approximately concentration-proportional increase in occupancy, together with the nearly unchanged peak positions, indicates that additional water predominantly populates the same types of local coordination environments rather than creating a different preferred water–PEEK contact geometry.

#### Hydration of Semicrystalline PEEK

To determine how crystalline morphology affects the local hydration of PEEK, RDFs and cumulative CNs were calculated separately for oxygen atoms located in the amorphous and crystalline regions of the semicrystalline models (Figure 4). The corresponding first-shell peak positions and coordination numbers are summarized in Table 1.

**Figure 4:**
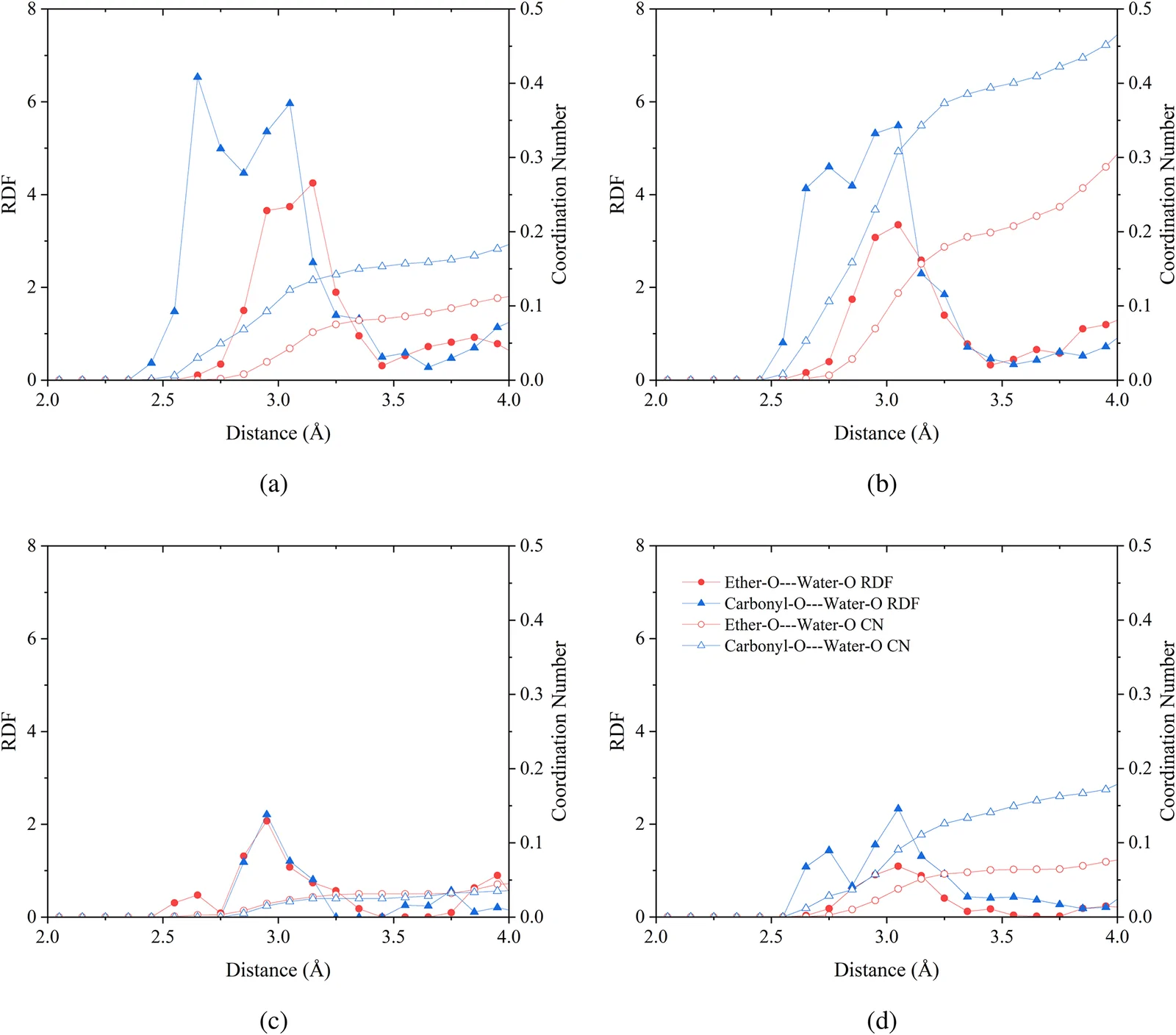
Radial distribution functions (RDFs) and cumulative coordination numbers (CNs) of water oxygen atoms around the ether and carbonyl oxygen atoms in the amorphous and crystalline regions of semicrystalline PEEK at 300 K. Results are shown for (a, c) 1 wt % and (b, d) 3 wt % water. (a, b) correspond to the amorphous region, and (c, d) to the crystalline region. All figures show the 2–4 Å region to emphasize first-shell features. Filled symbols represent RDFs (left axis), and open symbols represent cumulative coordination numbers (right axis). The cumulative CN is the average number of water oxygen atoms within distance *r* of a PEEK oxygen site; first-shell CNs were evaluated at the first minimum of each corresponding RDF.

The amorphous regions exhibited hydration behavior similar to that of the fully amorphous PEEK models. At 1 wt % water, the first-shell coordination numbers were 0.08 for ether oxygen and 0.15 for carbonyl oxygen. At 3 wt % water, these values increased to 0.19 and 0.44, respectively. Thus, carbonyl oxygen exhibited approximately 1.9–2.3 times the first-shell water occupancy of ether oxygen within the amorphous regions of S-PEEK. This result confirms that the preference of water for carbonyl sites observed in A-PEEK is retained within the noncrystalline regions of the semicrystalline morphology.

Hydration was substantially suppressed within the crystalline regions. At 1 wt % water, the ether and carbonyl coordination numbers decreased to 0.02 and 0.04, respectively, compared with 0.08 and 0.15 in the amorphous region. At 3 wt % water, the corresponding crystalline-region values were 0.06 and 0.11, compared with 0.19 and 0.44 in the amorphous region. Depending on the oxygen site and hydration level, the first-shell water occupancy in the amorphous region was therefore approximately 3.2–4.0 times that in the crystalline region.

Among water molecules assigned using the first minimum of corresponding RDF as the water-oxygen–PEEK-oxygen cutoff, approximately 84% and 83% had exclusively amorphous neighbors at 1 and 3 wt % water, respectively; approximately 6% had exclusively crystalline neighbors and 10–11% had both. Water without a PEEK oxygen neighbor within this cutoff accounted for approximately 9% and 14% of the total population, respectively. Regional water populations remained stable during the final 10 ps of equilibration across four replicas with randomized initial water placements. This supports stability over the sampled interval but does not establish long-time equilibrium partitioning. Assignment details and convergence results are provided in Section S2 of the Supporting Information.

Despite the pronounced differences in coordination number, the first-shell RDF maxima remained within approximately 2.65–3.15 Å for both morphologies. Crystallinity therefore altered the probability and extent of local hydration much more strongly than the characteristic water–PEEK contact distance. The lower coordination numbers in the crystalline domains are consistent with their dense and ordered molecular packing, which restricts the ability of water to approach the oxygen-containing groups. In contrast, the less densely packed amorphous and crystal–amorphous interfacial regions provide greater accessibility to these sites.

Taken together with the A-PEEK results in Figure 3, these findings demonstrate that PEEK hydration is governed by two complementary factors. Chemical functionality determines the relative local affinity for water, with carbonyl oxygen generally exhibiting greater first-shell occupancy than ether oxygen. Within the modeled morphology and sampled time scale, polymer packing controlled the accessibility of these sites, and the observed hydration of S-PEEK was concentrated preferentially within its noncrystalline regions. This heterogeneous water distribution provides a molecular basis for examining whether hydration preferentially modifies segmental mobility in the amorphous and crystal–amorphous interfacial phases.

The present periodic cell contains one stable crystalline domain. The model also lacks covalent chain connections between the crystalline and amorphous regions, which may affect interfacial load transfer and the constraint imposed on amorphous-chain mobility. At fixed crystallinity, subdivision into multiple smaller domains would increase the crystal–amorphous interfacial area and could reduce the quantitative hydration contrast observed here. At fixed water content and comparable local morphology, a larger system could improve sampling of water–PEEK contacts without necessarily increasing the coordination number, which is normalized per PEEK oxygen site. Larger cells could also accommodate morphological heterogeneity that changes site accessibility; the present replica analysis does not establish convergence with system size. The short trajectories also do not establish long-time water diffusion or equilibrium partitioning; slower water redistribution and polymer relaxation could alter the quantitative hydration and mechanical responses.

#### Comparison between Experimental and MD Simulation Studies

Previous experimental studies have indicated that absorbed water in PEEK includes mobile or self-associated populations and have provided limited evidence for strongly bound carbonyl–water complexes.^24,25^ This observation does not conflict with the present RDF and coordination-number results. The enhanced first-shell occupancy near carbonyl oxygen identifies a preferred local association site but does not establish that most absorbed water is carbonyl-bound or that individual contacts are long-lived. The present simulations therefore complement bulk spectroscopic measurements by resolving the chemical and morphological heterogeneity of hydration: water preferentially approaches carbonyl oxygen, while crystalline packing strongly limits the accessibility of both carbonyl and ether sites. This interpretation of local water–PEEK association is consistent with the combined experimental and ReaxFF study of Surve et al.;^26^ however, the present analysis does not quantify water–water self-association, and its structural conclusions are therefore limited to water–PEEK coordination.

### Effects of Hydration and Crystallinity on Molecular Mobility

The effects of hydration and crystallinity on PEEK molecular mobility were evaluated from the mean-square fluctuations of the polymer atoms, as shown in Figure 5. In the semicrystalline models, the MSF was calculated separately for atoms assigned to the amorphous and crystalline regions, allowing the mobility of each region to be compared with that of fully amorphous PEEK.

**Figure 5:**
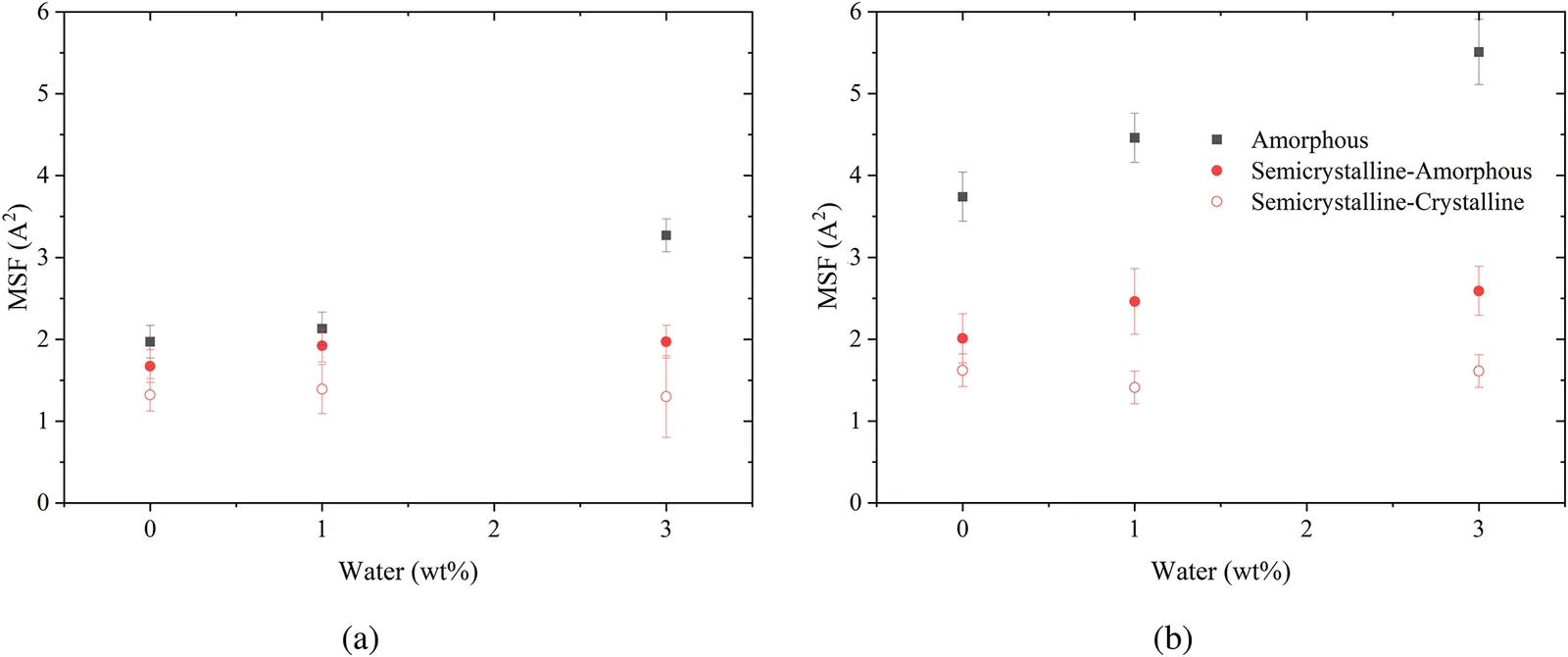
Mean-square fluctuation (MSF) of PEEK atoms as a function of water content at (a) 300 K and (b) 500 K. Black symbols represent the average MSF of the fully amorphous model. For the semicrystalline model, filled and open red symbols represent the amorphous and crystalline regions, respectively.

The fully amorphous PEEK models exhibited the greatest response to hydration. At 300 K, the average MSF increased from 1.97 Å^2^ in the dry model to 2.13 and 3.27 Å^2^ at 1 and 3 wt % water, respectively. At 500 K, the MSF increased from 3.74 Å^2^ in the dry system to 5.51 Å^2^ at 3 wt % water. Relative to the dry models, 3 wt % water produced absolute increases of 1.30 and 1.77 Å^2^ at 300 and 500 K, respectively, corresponding to relative increases of 66% and 47%. Thus, the absolute hydration-induced change was greater at 500 K, whereas the fractional increase relative to the dry polymer was greater at 300 K.

A different response was observed in semicrystalline PEEK. The amorphous regions of the semicrystalline models exhibited MSF values of 1.67–1.97 Å^2^ at 300 K and 2.01–2.59 Å^2^ at 500 K across the investigated hydration levels. These changes were smaller than those observed in fully amorphous PEEK. The crystalline regions consistently exhibited the lowest MSF, with values of 1.3–1.39 Å^2^ at 300 K and 1.41–1.62 Å^2^ at 500 K. Hydration therefore produced only a limited change in the mobility of the ordered domains over the water-content range investigated.

The reduced hydration response of the noncrystalline regions in semicrystalline PEEK indicates that their mobility is influenced by the surrounding crystalline morphology. Crystalline domains can restrict the conformational freedom of adjacent chains through topological and interfacial constraints, producing a less-mobile amorphous fraction near the crystal–amorphous interface.^27^ In addition, the regional-population, RDF, and coordination-number analyses collectively showed that the observed water population was concentrated within the amorphous regions, with substantially lower water accessibility and coordination inside the crystalline domains. The combination of restricted chain motion and heterogeneous water accessibility therefore limits the propagation of hydration-induced mobility throughout the semicrystalline structure. Consistent with this interpretation, the mean water-oxygen MSD at a lag time of 10 ps was lower in S-PEEK than in A-PEEK at both hydration levels and temperatures (Supporting Information, Section S3). Lower coordination around crystalline sites therefore did not imply greater overall water mobility; the smaller displacements are consistent with restricted pathways and reduced polymer rearrangement in the semicrystalline morphology.

These mobility results provide molecular context for the apparent density-derived *T*_g_ trend, although the hydration-induced differences in *T*_g_ were not resolved within the estimated uncertainty. The crystalline domains remained comparatively immobile at both hydration levels. Thus, absorbed water modifies molecular mobility primarily within accessible noncrystalline regions, while crystalline packing limits the magnitude and spatial extent of the plasticization response. This result is consistent with the general plasticization mechanism of absorbed water, in which small penetrant molecules increase the segmental mobility of accessible amorphous polymer chains and can decrease the glass-transition temperature.^28^

### Constrained Directional Modulus of Hydrated PEEK

The effects of hydration and crystallinity on the directional stiffness of PEEK were evaluated by applying uniaxial tensile deformation parallel (*z*) and perpendicular (*x*) to the crystalline chain direction (Figure 6). Because the two cell dimensions transverse to the loading direction were fixed, the deformation imposed zero transverse strain rather than zero transverse stress; the reported slopes are therefore constrained directional moduli rather than conventional Young’s moduli. The crystalline domain was separated from its periodic images by an amorphous region in all directions. Consequently, the calculated properties represent the effective response of the complete semicrystalline morphology rather than the intrinsic elastic constants of an isolated PEEK crystal.

**Figure 6:**
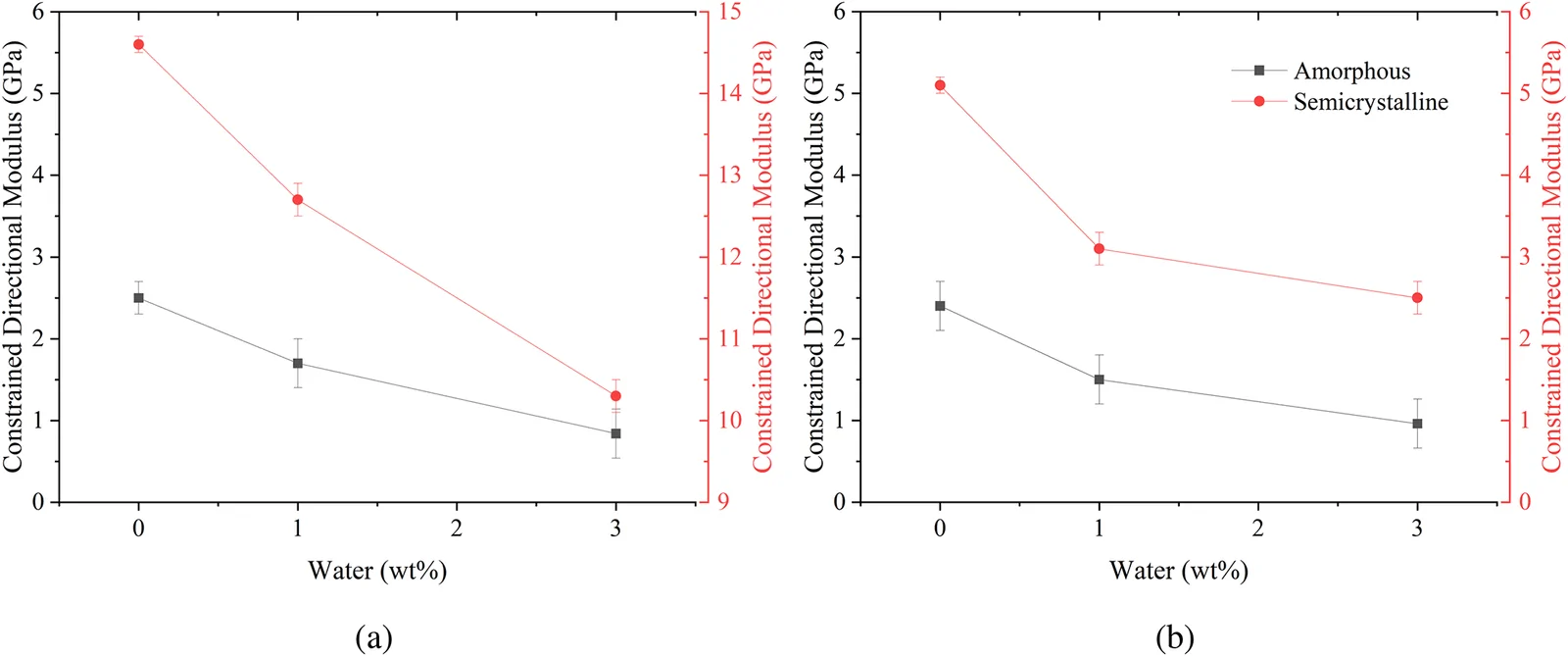
Constrained directional moduli of fully amorphous (A-PEEK) and semicrystalline PEEK (S-PEEK) containing dry, 1, and 3 wt % water at 300 K. Tensile deformation was applied (a) parallel to the crystalline chain direction (*z*) and (b) perpendicular to it (*x*). Black squares denote A-PEEK and correspond to the left axis; red circles denote S-PEEK and correspond to the right axis. Values were averaged over four independent simulations.

In the dry state, A-PEEK exhibited constrained directional moduli of approximately 2.5 and 2.4 GPa in the *z*- and *x*-directions, respectively. The similar values are consistent with the approximately isotropic mechanical response expected from the amorphous structure. Incorporation of the aligned crystalline domain increased the corresponding S-PEEK moduli to approximately 14.6 and 5.1 GPa. Thus, crystallinity increased the dry modulus by factors of approximately 5.8 parallel to the chain direction and 2.1 in the tested *x*-direction perpendicular to the chains.

The pronounced anisotropy of S-PEEK reflects the different molecular mechanisms resisting deformation. Loading parallel to the crystal chains directly engages the covalently bonded polymer backbones, whereas deformation in the tested *x*-direction depends more strongly on weaker interchain interactions and the mechanical coupling between the crystalline and noncrystalline regions. The longitudinal constrained directional modulus of the present semicrystalline model is influenced by deformation accommodated within its amorphous and interfacial regions, rather than reflecting only the intrinsic stiffness of the crystalline chains.^10^

The dry A-PEEK constrained directional modulus is comparable in magnitude to values from previous atomistic simulations, which reported an average Young’s modulus of 3.62 ± 0.94 GPa for amorphous PEEK.^10^ These numerical similarities should not be interpreted as direct validation because the present constrained moduli are not equivalent to Young’s moduli under zero transverse stress. This comparison should also be interpreted cautiously because the present S-PEEK value is an effective property of a finite crystalline domain embedded in amorphous PEEK, whereas the reported crystalline values describe an ideal periodic crystal.

Hydration reduced the modulus of both morphologies in both loading directions. For A-PEEK, the *z*-direction modulus decreased from approximately 2.5 GPa in the dry state to 1.7 and 0.85 GPa at 1 and 3 wt % water, respectively. The *x*-direction modulus similarly decreased from approximately 2.4 to 1.5 and 0.95 GPa. At 3 wt % water, these values correspond to reductions of approximately 66% and 60% in the *z*- and *x*-directions, respectively. The comparable softening in both directions is consistent with the absence of a preferred orientation in the amorphous structure.

For S-PEEK, the longitudinal modulus decreased from approximately 14.6 GPa in the dry state to 12.7 and 10.3 GPa at 1 and 3 wt % water, respectively, corresponding to a maximum reduction of approximately 29%. The *x*-direction modulus decreased more strongly, from approximately 5.1 to 3.1 and 2.5 GPa, corresponding to a reduction of approximately 51% at 3 wt % water. Hydration therefore weakened the tested *x*-direction response of S-PEEK more strongly than its chain-direction response.

This directional difference is consistent with the preceding hydration and mobility analyses. The regional water-population analysis showed that water was concentrated preferentially in the noncrystalline environments, while the MSF results demonstrated that the crystalline domains retained relatively low molecular mobility. The greater softening in the tested *x*-direction is consistent with plasticization of the amorphous and crystal–amorphous interfacial regions that contribute to load transfer in this direction. In contrast, the aligned covalently bonded chains within the crystalline domain continue to provide substantial longitudinal reinforcement. Accordingly, crystallinity does not eliminate hydration-induced softening, but it reduces its effect on the chain-direction stiffness of S-PEEK.

## Conclusions

Reactive molecular dynamics simulations were used to examine hydration in fully amorphous and semicrystalline PEEK containing 0–3 wt % water. Within the constructed models and sampled interval, water was concentrated in accessible amorphous environments and preferentially associated with carbonyl oxygen sites. Increasing water content raised the first-shell coordination numbers without substantially changing the characteristic water–PEEK separation, while crystalline packing strongly limited site accessibility.

Crystallinity also suppressed hydration-induced mobility and softening. At 3 wt % water, the MSF of fully amorphous PEEK increased by 66% at 300 K and 47% at 500 K, whereas the semicrystalline morphology showed smaller mobility changes. Its apparent *T*_g_ reduction of approximately 21 K was not resolved within the observed simulation variability. In dry semicrystalline PEEK, the constrained directional moduli were 14.6 and 5.1 GPa along the chain *z*-direction and the tested perpendicular *x*-direction, respectively; hydration reduced these values by 29% and 51%. The greater fractional reduction in the *x*-direction is consistent with plasticization of the noncrystalline and interfacial regions, while aligned crystalline chains retain substantial longitudinal reinforcement.

Overall, the simulated response was consistent with physical plasticization. The short simulation time scale does not establish resistance to long-term chemical degradation. Crystalline domains reduced water accessibility, constrained molecular motion, and preserved much of the chain-direction stiffness, supporting a molecular interpretation of the greater resistance of the modeled semicrystalline PEEK to short-time hydration-induced softening. These results provide a baseline without added catalysts for future investigations of catalyst-assisted hydrothermal degradation.

## Methods

### Atomistic Model

Two PEEK morphologies were constructed to investigate the effect of crystallinity on the early stage hydrothermal response: a fully amorphous model (A-PEEK) and a semicrystalline model (S-PEEK). The atomistic models are illustrated in Figure 7. The PEEK repeat unit, shown in Figure 7(a), contains three aromatic rings connected by two ether linkages and one ketone group.

**Figure 7:**
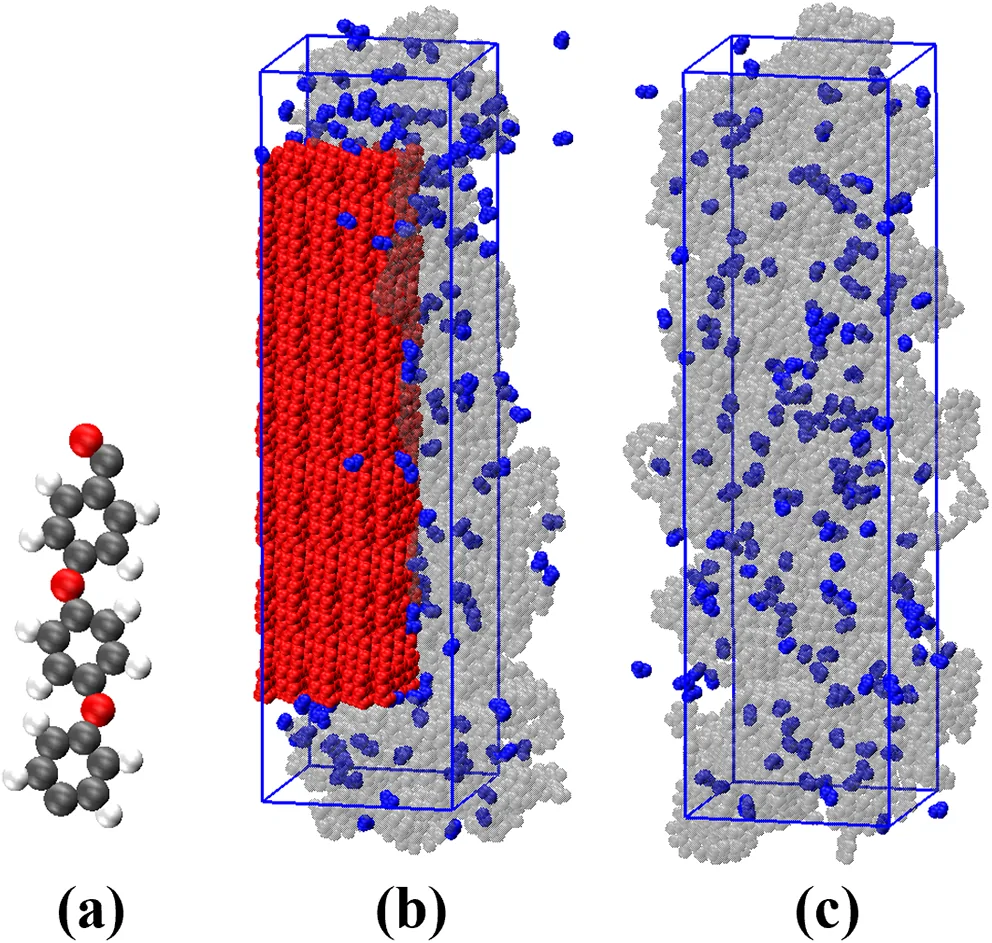
Atomistic models used in this study: (a) repeat unit of PEEK; (b) semicrystalline PEEK (S-PEEK) with a crystalline mass fraction of approximately 36 wt %, containing crystalline PEEK (red), amorphous PEEK (transparent gray), and 3 wt % water (blue); and (c) fully amorphous PEEK (A-PEEK) containing 3 wt % water.

The A-PEEK model was generated using Materials Studio by randomly packing 40 polymer chains, each containing 10 repeat units, into a three-dimensional periodic simulation cell. The initial structure was subjected to energy minimization followed by equilibration under the canonical (NVT) and isothermal–isobaric (NPT) ensembles at 300 K and 1 atm to eliminate unfavorable atomic contacts and obtain a stable amorphous structure. The equilibrated A-PEEK model contained 13,680 atoms and had simulation-cell dimensions of approximately 36.5 × 34.5 × 124.5 Å^3^ and an average equilibrated density of 1.23 gcm^–3^.

The S-PEEK model was constructed by embedding a crystalline PEEK lamella within an amorphous PEEK matrix. The crystalline lamella consisted of finite-length, hydrogen-capped PEEK chains, with separate amorphous PEEK molecules randomly placed around it. No covalent bonds connected the crystalline and amorphous chains; the two regions interacted through nonbonded contacts. The crystalline structure was generated from the experimentally determined unit cell of PEEK, with lattice parameters of *a* = 7.75 Å, *b* = 5.86 Å, and *c* = 10.0 Å.^29^ The unit cell was replicated 3 × 4 × 9 times along the *a*-, *b*-, and *c*-directions, respectively, producing an initial crystalline region with dimensions of approximately 23.25 × 23.44 × 90.0 Å^3^. The crystalline region accounted for approximately 36 wt % of the polymer. Several crystalline-domain sizes were evaluated during model development. The smaller domains tested lost crystalline order during equilibration; the selected domain was the smallest among those tested that retained crystalline order under the applied simulation conditions.

The polymer-chain direction was aligned with the *z*-axis. In the equilibrated configuration, the crystalline lamella was separated from its periodic image by approximately 32.0 Å of amorphous polymer along the *z*-direction. The corresponding separations were approximately 13.5 and 11.5 Å along the transverse *x*- and *y*-directions, respectively. The equilibrated S-PEEK model contained 13,695 atoms and had average simulation-cell dimensions of approximately 36.5 × 34.0 × 122.0 Å^3^. Periodic boundary conditions were applied in all three directions for both morphologies.

Three hydration levels were examined for each morphology: dry PEEK (0 wt % water) and PEEK containing 1 and 3 wt % water, where the water content was defined relative to the mass of the dry PEEK. The corresponding numbers of water molecules were 64 and 192 for the 1 and 3 wt % systems. Water molecules were randomly inserted into the accessible free volume of the equilibrated polymer structures while preventing atomic overlap using a minimum distance of 3 Å. Each hydrated configuration was subsequently subjected to energy minimization and equilibration using the procedure described in the next section. Representative configurations of S-PEEK and A-PEEK containing 3 wt % water are shown in Figure 7(b,c), respectively.

### Simulation Procedures

All MD simulations were performed using the Large-scale Atomic/Molecular Massively Parallel Simulator (LAMMPS), version 22 July 2025.^30^ Interatomic interactions were described using the ReaxFF reactive force field,^14^ which permits changes in atomic connectivity through a bond-order-dependent potential. The ReaxFF parameter set developed by Liu et al.,^31^ previously applied to dry PEEK by Pisani et al.,^10^ was used. Atomic charges were equilibrated at every time step using fix qeq/reaxff, with lower and upper taper radii of 0 and 10 Å, respectively, and a convergence tolerance of 10^–6^. Charge-equilibration parameters were read from the active ReaxFF parameter file. Periodic boundary conditions were applied in all three directions. Each initial configuration was subjected to energy minimization, followed by relaxation in the canonical (NVT) ensemble at 300 K for 50 ps and equilibration in the isothermal–isobaric (NPT) ensemble at 300 K and 1 atm for 100 ps. Isotropic pressure coupling was used during NPT equilibration. Equilibration was assessed from the stabilization of the temperature, potential energy, pressure, and density.

For determination of *T*_g_, each S-PEEK system was simulated from 200 to 600 K at intervals of 20 K. Temperature points were initialized independently. At each temperature, the system was equilibrated under the NPT ensemble for 100 ps, followed by an additional 50 ps of NPT simulation for density averaging.

Structural analyses were performed at 300 and 500 K. Four independent simulations were conducted for each morphology, hydration level, and temperature to evaluate statistical variability. For hydrated systems, the four replicas used different random seeds for initial water insertion. Following equilibration at the target temperature, production trajectories of 50 ps were collected under the NPT ensemble. Temperature and pressure were controlled using the Nosé–Hoover thermostat and barostat with damping constants 100 and 1000, respectively. An integration time step of 0.1 fs was used throughout the simulations.

### Structural Analyses

The glass transition temperature, *T*_g_, was estimated from the temperature dependence of the equilibrated system density. Separate linear regressions were fitted over 200–300 K and 500–600 K, and *T*_g_ was identified as the intersection of the fitted lines. Sensitivity to the fitting intervals and propagation of density uncertainty into the estimated *T*_g_ were evaluated as described in Section S1 of the Supporting Information.

Water–polymer interactions were characterized using radial distribution functions (RDFs) between water oxygen atoms and the ether and carbonyl oxygen atoms of PEEK. RDFs were calculated over distances up to 10 Å. The cumulative coordination number was obtained by integrating each RDF from zero to distance *r*; the first-shell coordination number was evaluated at the RDF’s first minimum for all investigated pairs. The coordination numbers represent the average numbers of water oxygen atoms surrounding each ether or carbonyl oxygen atom within the corresponding first coordination shell. These O···O distance-based quantities characterize local association and do not independently identify hydrogen bonds.

The local mobility of the polymer was quantified using the MSF of PEEK atoms. Center-of-mass translation of the polymer was removed before calculating the MSF. MSF values were averaged over all PEEK atoms for A-PEEK and separately over atoms assigned to the amorphous and crystalline regions for S-PEEK.

RDFs, first-shell coordination numbers, and MSFs were calculated from the equilibrated production trajectories at 300 and 500 K and averaged over four independent simulations.

### Mechanical Property Prediction

The elastic response of each PEEK system was determined by applying uniaxial strain independently along the *x*- and *z*-directions. In the semicrystalline models, the *z*-direction was parallel to the polymer-chain direction within the crystalline lamella, whereas the *x*-direction was perpendicular to the crystalline chains. The same coordinate directions were used for the amorphous models to facilitate comparison between the two morphologies. During tensile deformation, the loading direction was continuously expanded while the transverse simulation-cell dimensions were fixed, and the temperature was maintained at 300 K using a Nosé–Hoover thermostat. The resulting stress–strain slope therefore represents a constrained directional modulus, rather than a Young’s modulus obtained under zero transverse stress.

The simulation cell was continuously deformed at a constant engineering strain rate of 10^–5^ fs^–1^ (10^10^ s^–1^). The constrained directional modulus was obtained by linear regression of the axial stress–strain response over 0–5% strain, corresponding to 5 ps of deformation. Stress was sampled every 10 timesteps, and ten samples were averaged for each output written every 2000 timesteps, corresponding to sampling and output intervals of 1 and 200 fs, respectively. Four independent tensile simulations were performed for each morphology, hydration level, and loading direction. The reported directional moduli represent the effective elastic responses of the complete simulation cells; for S-PEEK, these include contributions from the crystalline, amorphous, and crystal–amorphous interfacial regions. They should therefore not be interpreted as the intrinsic elastic constants of the isolated crystalline phase.

## Supplementary Material



## Notes

Glass-transition fitting and uncertainty analysis; regional water-population convergence and coordination-number data; water mean-square-displacement analysis (PDF)
