# Supplementary material for: Crystallinity-Regulated Hydration, Molecular Mobility, and Mechanical Softening of Poly(ether ether ketone): A Reactive Molecular Dynamics Study
Source: ACS Omega. 2026 Oct 28;11(44):68925–34. doi: 10.1021/acsomega.6c08565 (PMC13756710; doi:10.1021/acsomega.6c08565)
Supplement: Supplementary file 1 [file ao6c08565_si_001.pdf]

Crystallinity-Regulated Hydration, Molecular Mobility, and Mechanical Softening of Poly(ether ether ketone): A Reactive Molecular Dynamics Study

Xiawa Wu*

Mechanical Engineering, Penn State Behrend, Erie, PA, USA

E-mail: xxw5228@psu.edu

S1. Glass-Transition Fitting and Uncertainty

The glass-transition temperature was estimated from the intersection of separate ordinary least-squares fits to the density data in the 200–300 K and 500–600 K regimes. Fitting-range sensitivity was evaluated using alternative intervals of 200–320/480–600 K and 200–280/520–600 K. Density uncertainty was propagated through 50,000 Monte Carlo realizations, independently perturbing each mean density using a Gaussian distribution with a standard deviation equal to its standard error and refitting both lines. The 2.5th and 97.5th percentiles of the resulting intersections defined approximate 95% uncertainty intervals. This procedure assumes independent errors between temperatures.

Table S1 summarizes the fitted transition temperatures, regression coefficients of determination, fitting-range sensitivity, and propagated uncertainty intervals. The primary fits yielded T_g values of approximately 435, 428, and 413 K for dry, 1, and 3 wt% water, respectively. The estimates

varied with the fitting intervals, and the propagated uncertainty was substantial, particularly for dry PEEK, where some perturbed fits produced nearly parallel lines and poorly determined intersections. Consequently, the lower fitted value at 3 wt% water indicates an apparent trend, but the present analysis does not resolve a hydration-induced shift in T_g.

Table S1: Density-derived glass-transition temperatures and fitting uncertainty for S-PEEK. Temperature intervals identify the nominal sampling ranges. The approximate 95% uncertainty intervals propagate density standard errors assuming independent errors between temperatures; they are not restricted to the simulated temperature range.

Quantity	Dry	1 wt%	3 wt%
Primary T_g (K)	434.8	428.4	413.4
Low-temperature R^2	0.974	0.963	0.895
High-temperature R^2	0.976	0.972	0.955
Alternative-range T_g, wider (K)	428.9	435.8	415.9
Alternative-range T_g, narrower (K)	440.8	384.8	401.8
Approximate 95% uncertainty interval (K)	[−51.5, 748.3]	[303.3, 498.5]	[344.3, 479.1]

Primary fitting intervals: 200–300 and 500–600 K. Wider intervals: 200–320 and 480–600 K. Narrower intervals: 200–280 and 520–600 K. The negative lower uncertainty bound for dry PEEK reflects instability of the line-intersection estimator, not a physically meaningful transition temperature.

S2. Regional Water Populations and Coordination Numbers

Regional water populations and coordination numbers were analyzed during the final 10 ps of NPT equilibration for four replicas with randomized initial water placements at each hydration level. Water molecules were classified using according to the PEEK oxygen neighbors within the first-minimum cutoff of the corresponding water-oxygen–PEEK-oxygen RDF: exclusively crystalline neighbors defined a crystalline environment, exclusively amorphous neighbors an amorphous environment, mixed neighbors an interfacial environment, and no neighbors an unassigned environment. Population fractions were normalized by the total water population; assigned-only fractions excluded unassigned water. Site-specific coordination numbers were calculated as the average number of water oxygen neighbors within the same cutoff per PEEK oxygen atom, separately for ether and carbonyl sites in each phase.

Figures S1 and S2 show the regional water population fractions during the final 10 ps for the

four replicas containing 1 and 3 wt% water, respectively. The populations remained stable over this interval, with amorphous coordination environments predominant across replicas. Table S2 summarizes the population fractions and site-specific coordination numbers, including their variability. These results support stability of the observed hydration pattern over the analyzed interval but do not establish long-time equilibrium partitioning.

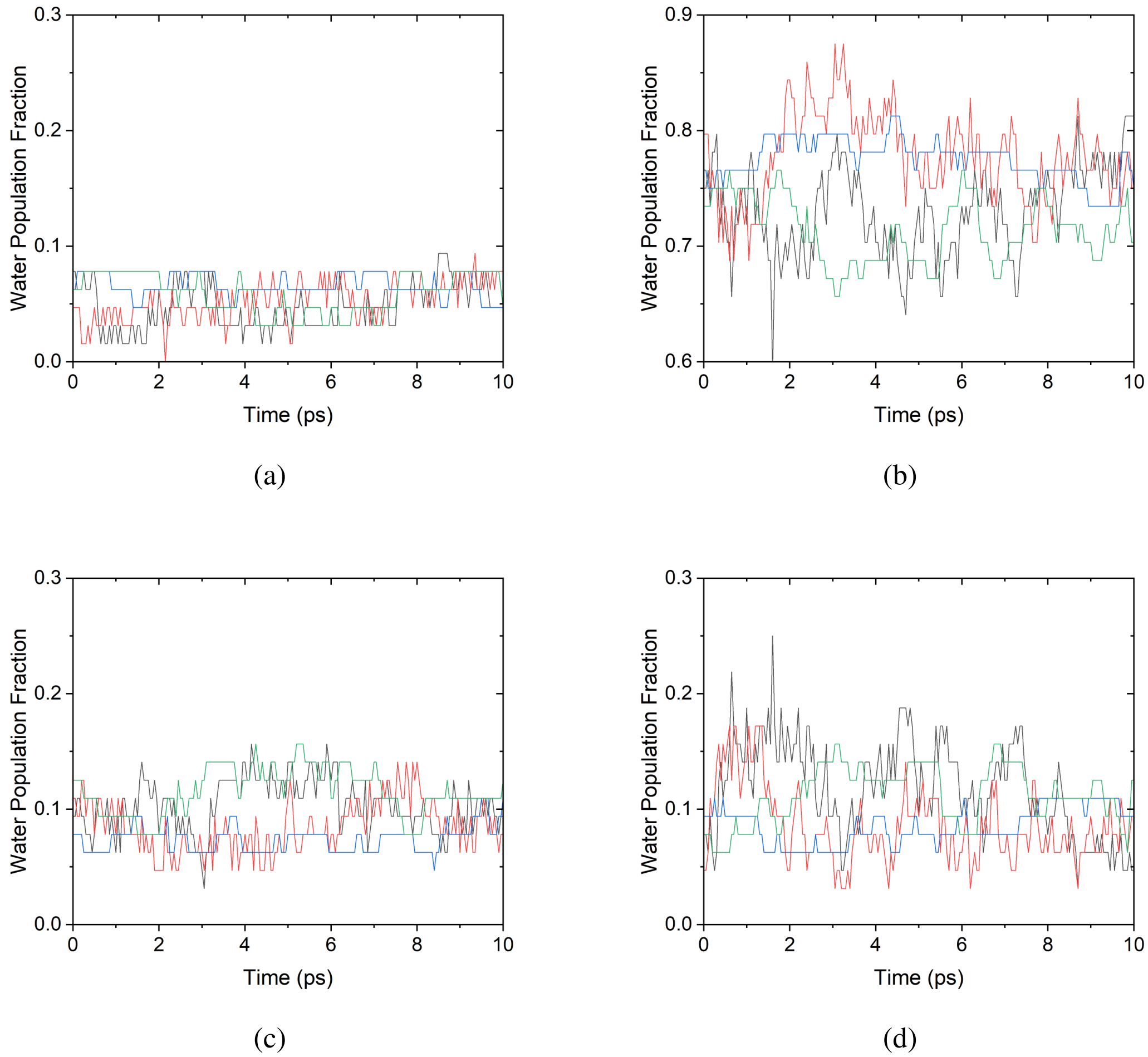

Figure S1: Water population fractions in (a) crystalline, (b) amorphous, (c) interfacial, and (d) unassigned coordination environments during the final 10 ps of NPT equilibration for S-PEEK containing 1 wt% water. Four colors in each subfigure correspond to four replicas with independently randomized initial water placements. Fractions are normalized by the total water population.

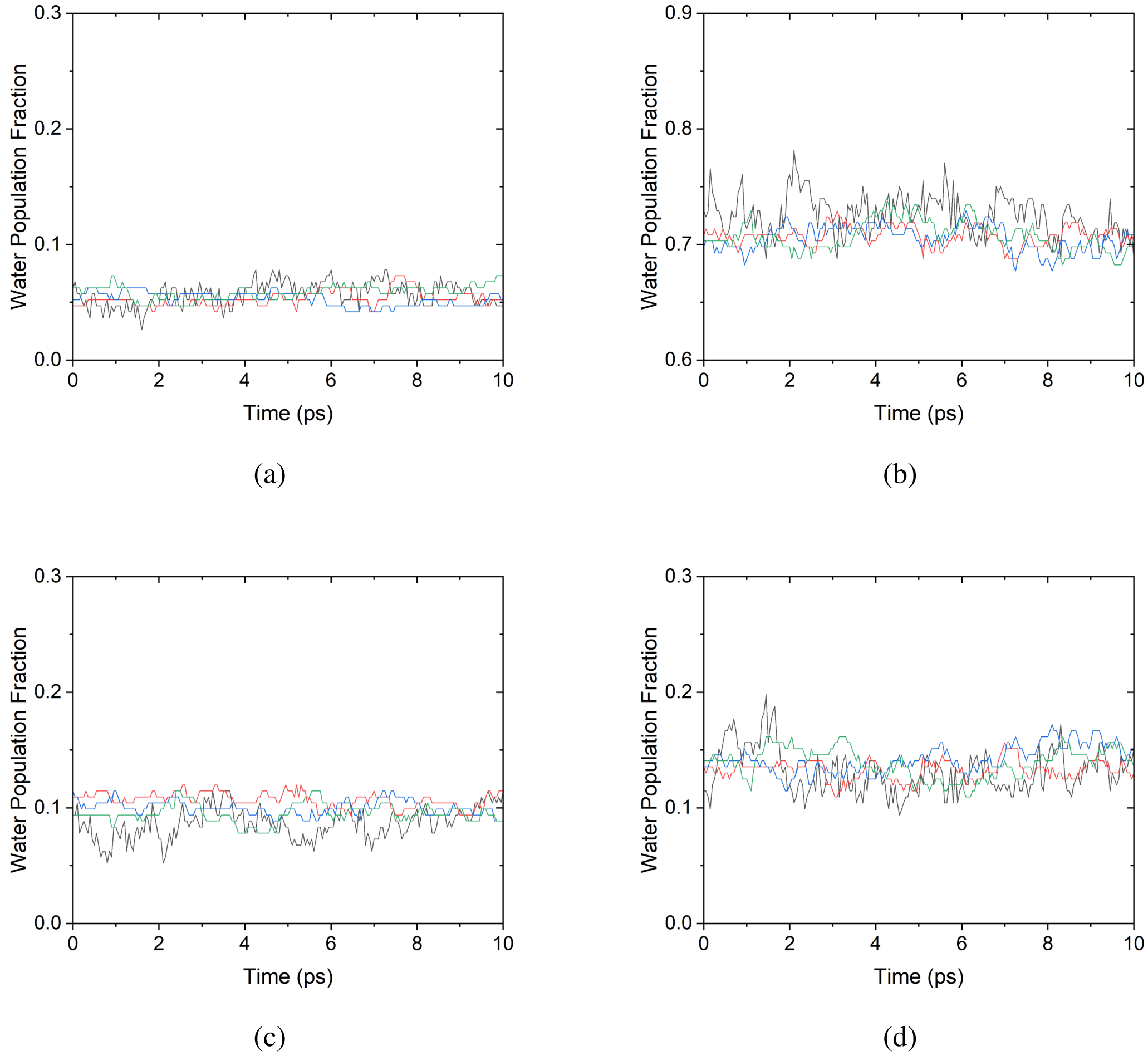

Figure S2: Water population fractions in (a) crystalline, (b) amorphous, (c) interfacial, and (d) unassigned coordination environments during the final 10 ps of NPT equilibration for S-PEEK containing 3 wt% water. Four colors in each subfigure correspond to four replicas with independently randomized initial water placements. Fractions are normalized by the total water population.

Table S2: Regional water population fractions and water–PEEK coordination numbers. Values are means ± standard deviations.

Quantity	1 wt% water	3 wt% water
Fractions relative to total water population		
Crystalline	0.06 ± 0.01	0.06 ± 0.01
Amorphous	0.76 ± 0.03	0.71 ± 0.02
Interfacial	0.09 ± 0.02	0.09 ± 0.01
Unassigned	0.09 ± 0.03	0.14 ± 0.02
Fractions relative to assigned water population		
Crystalline	0.06 ± 0.02	0.06 ± 0.01
Amorphous	0.84 ± 0.02	0.83 ± 0.01
Interfacial	0.10 ± 0.03	0.11 ± 0.01
Coordination number per PEEK oxygen atom		
Crystalline ether	0.02 ± 0.00	0.06 ± 0.01
Crystalline carbonyl	0.04 ± 0.01	0.11 ± 0.01
Amorphous ether	0.08 ± 0.01	0.19 ± 0.01
Amorphous carbonyl	0.15 ± 0.01	0.44 ± 0.02

Assigned fractions exclude water molecules without a PEEK oxygen neighbor. Coordination numbers use the same cutoff. Population fractions may not sum to unity because of rounding.

S3. Water Mean-Square Displacement

Water translational mobility was evaluated from the mean-square displacement (MSD) of water oxygen atoms in A-PEEK and S-PEEK containing 1 and 3 wt% water at 300 and 500 K. For each replica, squared displacements were averaged over water oxygen atoms and all available time origins at each lag time. No additional polymer-motion subtraction was applied. Results were averaged across four replicas, with standard deviations describing between-replica variability. Values at a common lag time of 10 ps were used for numerical comparison; diffusion coefficients were not extracted.

Figure S3 compares the water MSD curves, and Table S3 summarizes the values at a lag time of 10 ps. At this lag, the mean MSD was lower in S-PEEK than in A-PEEK by approximately 54% and 44% at 300 K for 1 and 3 wt% water, respectively; the corresponding differences at 500 K were 21% and 23%. Lower crystalline-site coordination therefore did not correspond to greater overall water mobility. Most assigned water in S-PEEK occupied amorphous coordination environments, and coordination numbers measure occupancy rather than interaction strength. The

smaller displacements are consistent with restrictions imposed by the crystalline morphology and reduced rearrangement of the surrounding polymer, although these effects were not separately quantified.

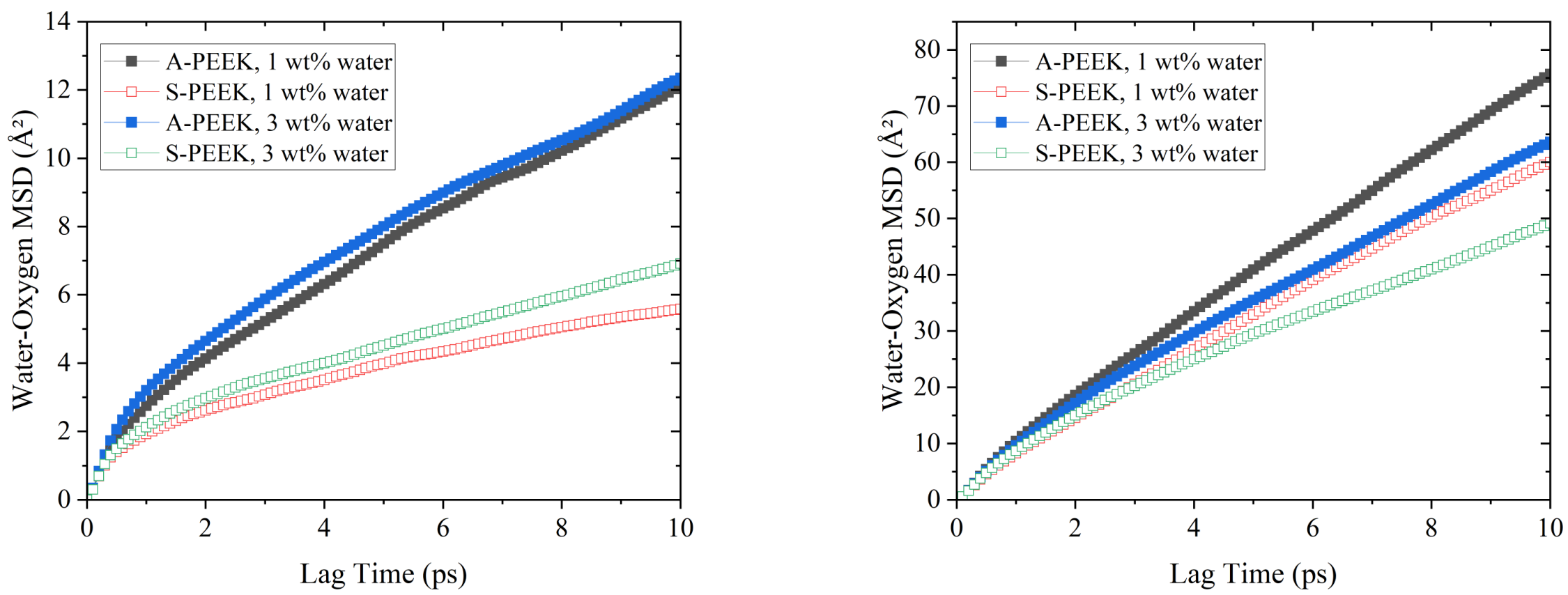

(a) (b)

Figure S3: Water-oxygen MSD as a function of lag time for A-PEEK and S-PEEK containing 1 and 3 wt% water at (a) 300 K and (b) 500 K. Curves represent means across four replicas

Table S3: Water-oxygen MSD at a lag time of 10 ps. Values are means ± standard deviations across four replicas.

Temperature (K)	Water (wt%)	A-PEEK MSD ($Å^2$)	S-PEEK MSD ($Å^2$)
300	1	12.12 ± 1.23	5.59 ± 0.53
300	3	12.35 ± 3.23	6.92 ± 2.40
500	1	75.73 ± 5.32	60.03 ± 10.97
500	3	63.63 ± 5.34	49.14 ± 13.80
